# The Availability and Nutritional Adequacy of Gluten-Free Bread and Pasta

**DOI:** 10.3390/nu10101370

**Published:** 2018-09-25

**Authors:** Beatrice Allen, Caroline Orfila

**Affiliations:** School of Food Science and Nutrition, University of Leeds, Leeds LS2 9JT, UK; beatriceallen@hotmail.co.uk

**Keywords:** coeliac disease, celiac disease, gluten, gluten-free diet, fortification, micronutrient, cost

## Abstract

Management of coeliac disease (CD) requires the removal of gluten from the diet. Evidence of the availability, cost, and nutritional adequacy of gluten-free (GF) bread and pasta products is limited. GF flours are exempt from UK legislation that requires micronutrient fortification of white wheat flour. This study surveyed the number and cost of bread and pasta products available and evaluated the back-of-pack nutritional information, the ingredient content, and the presence of fortification nutrients of GF bread and pasta, compared to standard gluten-containing equivalent products. Product information was collected from four supermarket websites. Standard products were significantly cheaper, with more products available than GF (*p* < 0.05). GF bread products were significantly higher in fat and fiber (*p* < 0.05). All GF products were lower in protein than standard products (*p* < 0.01). Only 5% of GF breads were fortified with all four mandatory fortification nutrients (calcium, iron, nicotinic acid or nicotamide and thiamin), 28% of GF breads were fortified with calcium and iron only. This lack of fortification may increase the risk of micronutrient deficiency in coeliac sufferers. It is recommended that fortification legislation is extended to include all GF products, in addition to increased regulation of the nutritional content of GF foods.

## 1. Introduction

Coeliac disease (CD) affects 1% of the population in Europe [1,2]. Gluten is a collection of storage proteins present in major cereals such as wheat, barley, and rye, which compromise the main components of common UK foods, bread and pasta [3]. When consumed, gluten proteins trigger an immune-mediated enteropathy, leading to intestinal damage in genetically pre-disposed individuals. Management of CD requires exclusion of dietary gluten, which proves a challenge for CD sufferers due to the presence of gluten in many food products [4]. Cereals and their products contribute important dietary nutrients, including high levels of protein, fiber and B vitamins such as niacin and thiamin. In addition, white wheat flour is fortified by law in the UK with calcium, iron, nicotinic acid or nicotamide and thiamin. Thus, removal of wheat products from the diet may result in nutrient insufficiency in coeliac diets. 

The manufacturing of gluten-free (GF) products is challenging, as gluten contributes vital structural, rheological, and organoleptic properties to bread and pasta [3]. Currently, no direct substitute for gluten is available and a combination of refined unfortified cereal flours (e.g., maize and rice), hydrocolloids (e.g., hydroxypropyl methylcellulose) and proteins (e.g., egg white) are used to make GF products, which are often unpalatable [5]. There is inadequate evidence on the impact these foods have on the health of consumers; however, studies have demonstrated a strong impact of a GF diet on diet-related quality of life, affecting in particular their ability to eat socially and outside the home [6].

In addition to a reduced nutritional profile of GF foods, consuming a GF diet (GFD) has financial and psychological effects on CD patients. The inability to purchase affordable food easily may result in CD patients experiencing higher levels of depression and increased psychological stress regarding food consumption, especially in social situations [7].

The UK National Diet and Nutrition Survey (NDNS) reported white bread was the most commonly consumed carbohydrate source, and pasta consumption is increasing [8]. If CD patients are directly exchanging standard products for GF equivalents, GF products need to be of a similar nutritional standard. Due to the high intakes of bread, UK manufacturers are required by the Bread and Flour Regulations (1998) to fortify bread flours with calcium, iron, nicotinic acid or nicotamide and thiamin [9] to prevent micronutrient deficiencies in the population. The Science Advisory Committee on Nutrition advised in 2017 mandatory folic acid fortification of standard flour to prevent anencephaly and spina bifida in developing fetus [10], although currently no changes to legislation have been made. GF flours and bread are exempt from these regulations, the reasons for which are unclear.

It is generally considered that GF foods are less nutritionally adequate than standard products, although evidence is limited. Fry et al. (2018) observed higher levels of fat, sugar and salt in UK GF foods compared to standard foods, although the study focusses solely on front-of-pack macronutrient levels [11]. Thompson (1999) found only 9% of US GF bread products were fortified with thiamin, riboflavin, and niacin [12]. Thompson (2000) reported iron fortification in 23% of GF breads and no fortification in US GF pasta products [13]. Recent studies have demonstrated low intakes of micronutrients in coeliac diets [14,15,16,17], this may be of particular concern in children [17].

The Department for Environment Food & Rural Affairs (2013), supported by manufacturing industry representatives and public health officials, confirmed its retention of the existing fortification legislation. According to the review, fortification of foods is cheap, convenient for manufacturers and nutritionally beneficial for the general population. There is no apparent reason GF products are not included in fortification legislation. This lack of fortification of GF foods may increase the risk of micronutrient deficiency and have severe health consequences for CD patients and consumers who choose to avoid gluten. However, recent studies have shown that CD patients may accumulate heavy metals, due to higher metaloprotein expression in enterocytes [18] so any fortification strategy must be carefully considered.

This study aims to examine the availability, cost and nutritional composition of GF bread and pasta, specifically addressing the macronutrient content and micronutrient fortification of these foods. 

## 2. Materials and Methods

This study was a cross-sectional survey of the availability, cost, ingredient labels and nutritional values of standard and GF products available to buy in the four major UK supermarkets through online retailer websites. Products selected for the GF data had a clear GF declaration or description on the packaging. For each available GF product, a standard gluten-containing product of similar characteristics was selected e.g., a GF white roll matched with a standard white roll. The four product categories selected were white bread, brown bread, seeded bread and white pasta, to reflect common UK consumption habits. A list of products identified through the survey is available as supplementary material, and includes information on brand, cost, back-of-pack energy, macronutrient and salt content, and presence of fortification nutrients.

### 2.1. Data Collection

#### 2.1.1. Search Strategy

To represent the majority of purchasing options available to UK consumers, the four biggest supermarkets were chosen; Tesco, Sainsbury’s, ASDA and Morrisons. Collectively, these supermarkets had a 73.2% market penetration in the UK grocery market in 2015. High end supermarkets and specialist health shops, such as Waitrose and Holland & Barrett, were excluded from the study because of the higher price range of products and smaller numbers of stores, which may not be accessible to all coeliac consumers. Discount retailers such as Aldi and Lidl that display no product or nutritional information online were excluded from the study.

The data was collected between September 2017 and December 2017. The product name, the cost (£/100 g), the ingredient label and the nutritional information (per 100 g) were recorded. Values relating to micronutrient levels were recorded where available, although this was limited in many products. Branded products found in multiple supermarkets were recorded once in the study. Where the same products were available in multiple formats (e.g., branded bread at 400 g and 800 g), only one format was selected for the study. 

Two search methods were used; typing in general product names (e.g., white bread) into the search bar function on the supermarket websites and secondly by selecting product categories, as defined by the supermarkets e.g., category: bread, sub-category: white bread. Both search methods returned relevant products; however, both relied on the efficiency of the website and the correct labelling of the products and food categories by the supermarkets. Using both methods of searching for products and cross-referencing the resulting products in each category allowed the relevant products to be made available to the study. This was necessary, as using the search bar often returned irrelevant products, or the category selection did not include all available GF products. Search terms for GF products included GF, coeliac and free-from, and was used in addition to selecting the GF product category on the supermarket websites. 

#### 2.1.2. Food Categories 

Products included in the evaluation for bread included whole loaves, sliced loaves, rolls (and their regional names e.g., baps). Bread products not included in the evaluation were part-baked breads, bread flours, crackers, wraps, pittas, bagels, crumpets, English muffins, 50/50 bread (half white/half brown), and naan breads as these often did not have GF equivalents. Brown bread and seeded bread products were recorded separately owing to the impact that seeds may have on the nutritional content. The brown bread category included any bread that was labelled as wholemeal or brown. Any bread product with labelling such as grains, granary, malted or any labelling referring to a seed were categorized as “seeded” and included in the “seeded bread” data set. 

Pasta products used for the standard products evaluation included dry white pasta. Nutrient values were recorded as to the dry weight to ensure that products were comparable. Standard pasta products not recorded in the review included wholemeal pasta, canned pasta, pasta ready meals, gnocchi, 50/50 pasta, and fresh pasta. GF pasta products selected were direct substitutes of white pasta, therefore products such as pea pasta or red lentil pasta were not included in the study, as they do not directly mimic standard white pasta characteristics. The list of all the products included in the survey can be found Table S1.

#### 2.1.3. Nutritional Content

The back-of-pack nutritional information was recorded for each product, including energy (kJ) per 100 g, energy (kcal) per 100 g and the following nutrients recorded in g per 100 g; fat, saturated fat, carbohydrates, sugars, fiber, protein, salt. Other nutrients were recorded where the information was provided, including calcium (mg), folic acid (µg), iron (mg), niacin (mg), omega-3 fatty acids (g), riboflavin (mg) and thiamin (g). The mean value and standard deviation was calculated and a graphical representation was created. The percentage of the contribution to the Reference Nutrient Intake (RNI) per portion was calculated using the mean value for 100 g of bread and 75 g of pasta for standard and GF products. The nutrient content of all the products included is listed in Table S1.

#### 2.1.4. Ingredient Labels 

The ingredient label was recorded for each product and a detailed list of the individual ingredients was compiled. Ingredients that had the same origins, for example, rice flour, rice starch, rice flakes etc. were grouped together under an appropriate collective term, e.g., rice. The functions of the ingredient (e.g., preservative) and the general nutritional contribution (e.g., protein) were recorded. The total number of ingredients that occurred in each product was calculated, in addition to the mean number of ingredients for each product category. The percentage of standard and GF foods containing each ingredient was calculated. Word it Out (2018) online software was used to analyze the frequency in which ingredients were found on labels. The list of ingredients for all the products included in this survey can be found in Table S2 (standard products) and Table S3 (GF products).

#### 2.1.5. Fortification of Products 

The fortification count of GF products was established by examining each ingredient label for the following nutrients; calcium carbonate, iron, niacin, and thiamin. The mean nutrient content was calculated for products where values were given, and the resulting information used to calculate the contribution to the RNI. The estimated contribution to the RNI for the standard breads was calculated using the minimum fortification values for each micronutrient per 100 g of wheat flour (the Bread and Flour Regulations, 1998). The presence of fortification for each product in the survey was recorded in Table S1.

### 2.2. Calculations and Statistical Analysis 

Microsoft Excel was used to calculate the mean values, standard deviation for this study and perform all statistical tests for this study. Mean and standard deviation for each product category were calculated, and their differences assessed using an f-test, followed by a *t*-test (two-tailed distribution and two-sample unequal variance (heteroscedastic) settings). Statistically significant differences between means were considered when *p* < 0.05. 

## 3. Results

### 3.1. Availability of GF Bread and Pasta 

GF breads and pasta were available from all four websites and each supermarket sold an own-brand GF range. Often the same brands e.g., Genius and Schar were available from all websites, thus the products available may be repetitive and indicate a low level of consumer choice.

**Figure 1:**
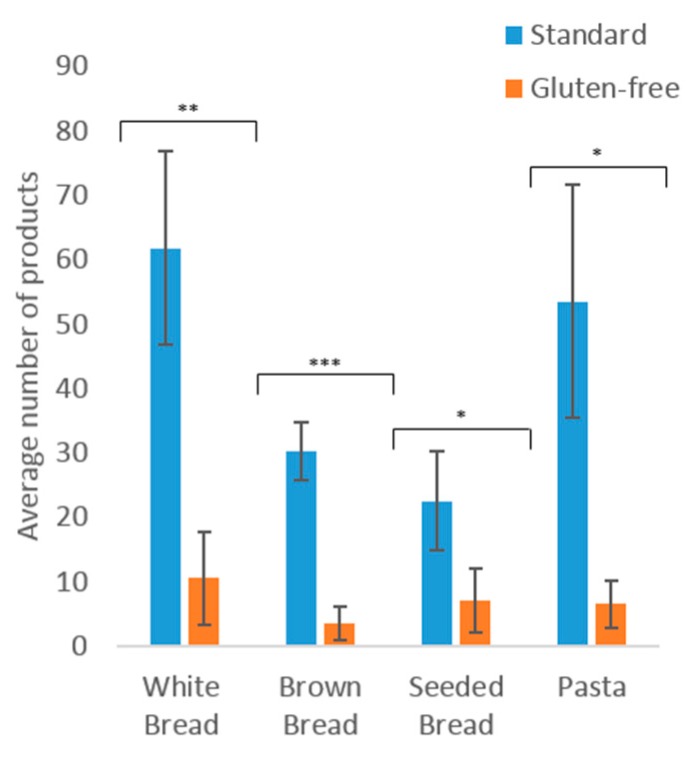
The average number of standard and gluten-free products available in four major UK supermarket websites. Key: * = *p* < 0.05, ** = *p* < 0.01, *** = *p* < 0.001.

As shown in Figure 1, there were significantly more standard products available than GF products across all categories. The mean number of standard products available ranged between 23 and 62, while the mean available GF products ranged between 4 and 11. There was a significant difference between the number of GF white breads available, compared to the standard (*p* = 0.003). The lower number of GF brown breads compared to the standard was significant (*p* ≤ 0.001). This is indicative of the limited choice available for GF consumers. GF pasta had significantly lower numbers of products available than standard products (*p* = 0.012). There were 83–88% fewer GF white and brown breads and pasta products available compared to standard products. There were 69% fewer GF seeded breads than the standard equivalent. 

A total of 110 products was used in the cost and nutritional analysis section of this study. All the available GF products (*n* = 49) on the websites were used and compared to equivalent standard products (*n* = 61). 

### 3.2. Product Cost

**Figure 2:**
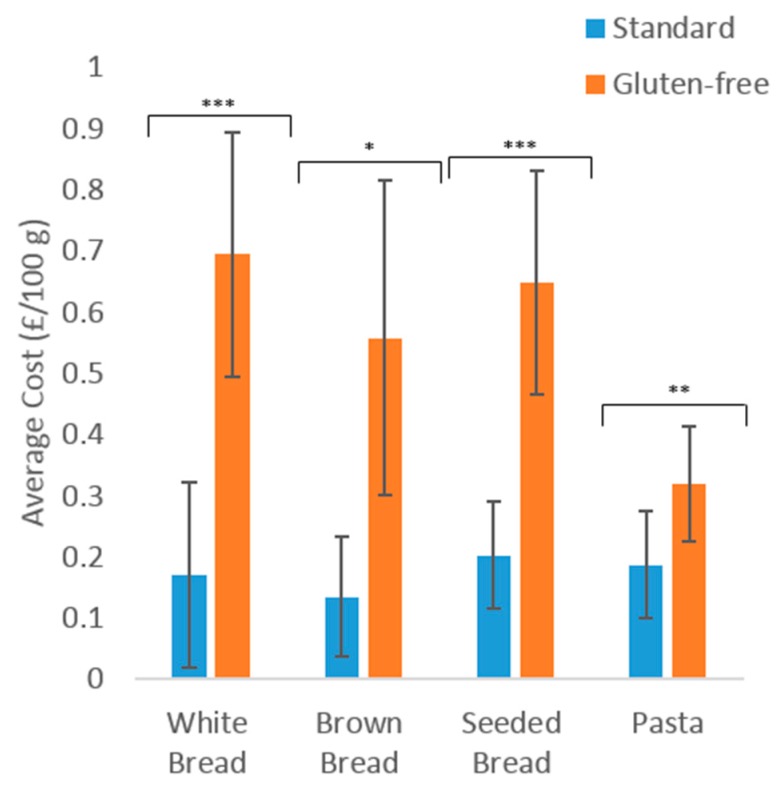
The average cost (£/100 g) of standard and gluten-free products available in four major UK supermarket websites. Key: * = *p* < 0.05, ** = *p* < 0.01, *** = *p* < 0.001.

The average cost of standard and GF products is shown in Figure 2. GF products were significantly more expensive across all four product categories than the standard equivalents. A highly significant difference was observed between the higher cost of GF pasta and standard pasta (*p* = 0.004). An extremely significant difference was observed in the cost of GF white and seeded breads compared to the standard equivalents (*p* < 0.001). When compared to standard products, an increase in the average cost of GF products was observed in white (+307%), brown (+314%) and seeded (+220%) breads. The cost of GF pasta increased by 70% compared to standard pasta. 

### 3.3. Ingredient Labels and Nutritional Adequacy of Products

**Figure 3:**
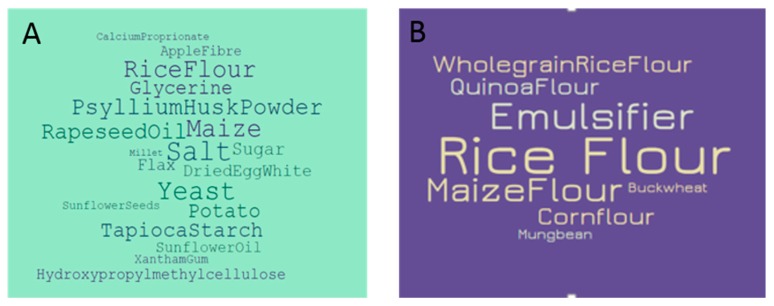
Key ingredients used in gluten-free (**A**) bread products and (**B**) pasta products. Ingredients frequently used in multiple products are indicated by larger font sizes. For a full list of ingredients, please refer to supplementary material.

The total number of different ingredients used for GF bread was 98 and 44 for standard breads. A list of ingredients for all products in this survey can be found in Table S2 (standard products) and Table S3 (GF products). On average, eight different ingredients were used for GF pasta and one for standard pasta. The average number of ingredients used in standard bread products was 14 compared to 20 for GF bread. The higher number of ingredients was not associated with higher cost. The most common GF ingredients used are shown in Figure 3, with common ingredients being cereal flours, plant fibers and emulsifiers. 

**Figure 4:**
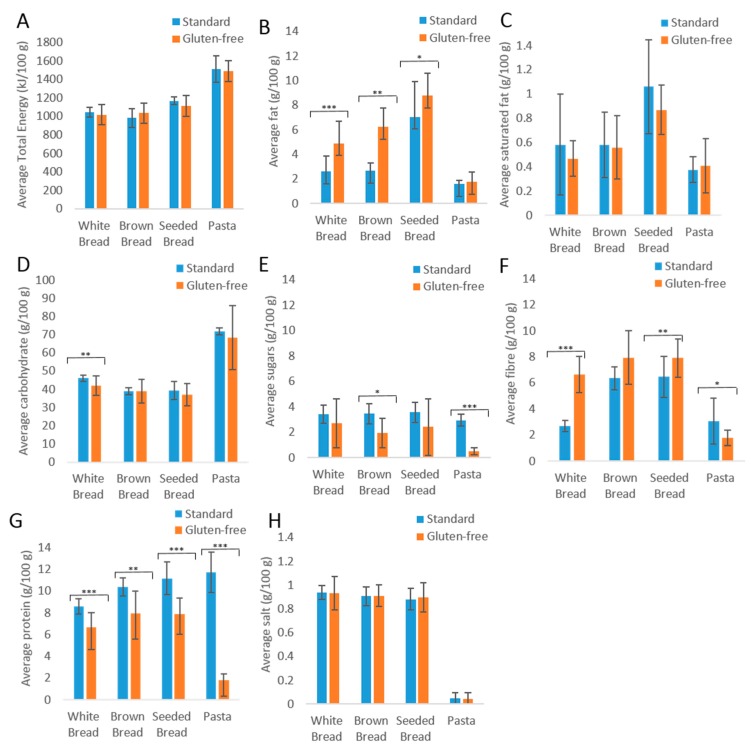
The average nutritional values of standard and gluten-free products for (**A**) total energy; (**B**) fat; (**C**) saturated fat; (**D**) carbohydrate; (**E**) sugars; (**F**) fiber; (**G**) protein; (**H**) salt. Key: * = *p* < 0.05, ** = *p* < 0.01, *** = *p* < 0.001.

The nutrient content per portion of standard and GF products and their contribution to RNI is shown in Figure 4. The average total energy, saturated fat, and salt values (Figure 4A,C,H) were similar between standard and GF products. Carbohydrate values were significantly lower in GF white bread (*p* = 0.004); however, the carbohydrate values were similar and not statistically different for other product types (Figure 4D). 

Standard products contained higher levels of sugar (Figure 4E) than GF products, significantly so in brown bread (*p* = 0.041) and pasta (*p* = <0.001). The RNI is the daily intake needed to satisfy the nutritional requirements of the majority (97.5%) of the population, as shown in Table 1. The sugar content in one portion of standard brown bread and pasta as a contribution to the RNI is 4% and 2% respectively (Table 1). 

Fiber was higher in all GF bread products (Figure 4F), significantly in white bread (*p* ≤ 0.001) and seeded bread (*p* = 0.008). A portion of GF bread contributes 22–26% of the fiber RNI, compared to standard breads providing 9%–21%. Standard white bread contained the lowest levels of fiber at 3 g per 100 g. The fiber content of GF pasta is significantly lower than standard pasta (*p* = 0.039), contributing 50% less fiber to the RNI than the standard, at 4% and 8% respectively. 

As shown in Figure 4B, GF products contain significantly higher fat levels in white (*p* ≤ 0.001), brown (*p* = 0.006) and seeded (*p* = 0.038) breads, with a minimal difference noted in pasta products. A portion of GF bread could contribute 7%–13% of the fat RNI, compared to standard products ranging between 4% and 10%. 

Protein (Figure 4G) was significantly lower in all GF products, especially in white and brown breads (*p* ≤ 0.001) and pasta (*p* ≤ 0.001) when compared to standard products. All GF products contribute between 9%–16% of the RNI per portion, compared to standard products which contribute 17%–22% of the RNI. 

### 3.4. Fortification of Bread and Pasta 

All white bread is fortified with calcium carbonate, iron, nicotinic acid or nicotamide and thiamin in accordance with UK law [19]. Standard brown (30%) and seeded (94.7%) breads contained fortified wheat flour although it is assumed that the use of wholemeal flour and seeds may increase the micronutrient content of these products. In total, 10% of standard bread products contained added folic acid. 

Our analysis showed that only 28% of GF breads were fortified with calcium carbonate and iron, and only 5% of the total GF breads were fortified with all four fortification minerals, in addition to folic acid and riboflavin. Only two of the 14 GF manufacturers, Genius and Warburtons, fortified their products. None of the standard and GF pasta products surveyed was fortified. 

Fortification of bread with nutrients contributes substantially to the RNI, especially that of calcium (23.5%) and thiamin (24%–30%) (Table 2). These results demonstrate that where fortification is present, a considerable percentage of the RNI can be achieved in one portion of bread. Most GF products on the market are not contributing to this intake.

## 4. Discussion

The results of this study clearly show that CD sufferers are at risk of nutritional inadequacy owing to three main factors: (1) the low availability and high cost of commercial GF alternatives to bread and pasta; (2) the reduced nutritional quality of GF products compared to their equivalent gluten-containing staples; (3) the widespread lack of fortification.

### 4.1. Availability and Cost

These results demonstrate significantly lower numbers of GF products were available, compared to standard products. However, the true number of available products may be considerably lower, as the websites list GF products as sold throughout the country. It is likely that the actual number of in-store products will relate to the size of the supermarket and thus, in smaller supermarkets the number of GF products may be further restricted. This may considerably impact CD patients living in rural areas or those with access to only one supermarket. The limited number of products and potentially limited access to food may contribute to increased anxiety and depression surrounding food choice reported in CD patients [7]. However, global sales of GF food grew by 12.6% in 2016, with the market expected to increase [20]. This could be due to increased awareness of CD and a consumer perception that GF products are healthier. Despite the limited number of GF products currently available, the expansion of the GF product market can only contribute positively to coeliac food choice. 

The results also showed that GF products are more expensive, in line with previous findings by Fry, Madden [11] which found GF products were on average 159% more expensive than the standard equivalents. Generally, standard bread loaves in the UK weigh 800 g, thus the average price of a standard white bread loaf is £1.35, compared to the higher GF cost of £5.52 of comparable weight.

The results of this study demonstrate that in addition to the limited number of products available to the consumer, the cost of GF products is significantly more expensive than standard products. The increased economic burden of CD and the reduced availability of products may increase the likelihood of some CD patients being unable to comply with a GFD. 

### 4.2. Nutritional Profile According to Back-of-Pack Labelling

The results indicated that GF products tend to contain more fiber and fat, but less sugar and protein. The higher fiber content of GF breads may be attributed to the frequent use of plant fibers in GF products (Figure 3). Ingredients such as psyllium husk powder, cellulose polymers such as hydroxypropyl methylcellulose and apple fibers are commonly used to in GF manufacturing, as confirmed by the findings of Capriles and Areas [5]. The NDNS [21] reported that cereals and cereal products provide the largest source of dietary fiber, with only 9% of adults aged 19 to 64 years achieving the RNI of 30 g per day. CD patients following a GFD consume inadequate fiber levels, especially women, with intakes of 13.7 g per day [15] and 12 g per day [14] observed in UK dietary studies (fiber expressed as non-starch polysaccharides). There is limited scientific evidence of the effects these products have on the digestive system, but recently a study has shown that a subset of CD patients is sensitive to fermentable fibers [22]. Understanding these effects is important, especially as the digestive mechanism of CD patients may differ to that of the general population, as CD is an abnormal immune response to a food component. 

Wild et al. found that CD patients following a GFD consumed significantly higher intakes of fat at 68.4 g per day and 93.2 g per day for women and men respectively, the latter exceeding the RNI of fat of <70 g per day [15]. These levels are higher than those observed by Kinsey et al. [14] who reported mean fat intakes of 59 g per day, which equates to 85% of the RNI for fat. Cereals account for a substantial 23% of UK protein intake [23]. Both studies found that coeliac protein consumption exceeded the RNI of 70 g per day with mean values of 74.1 g and 75 g of protein consumed per day, respectively. Therefore, while GF products are significantly lower in protein, this is unlikely to put CD sufferers at risk of protein deficiency. These results are supported by those of Fry et al. who compared the front-of-pack nutritional content of GF and standard products for example breads, cereals, pizza, and crackers [11]. Their study found that GF breads contained higher fat and fiber, and lower protein values than standard breads. Their study has several limitations, including the unexplained exclusion of GF products containing xanthan gum and egg white replacer, which are common in GF foods (Figure 3A). Although there is a statistically significant difference in the sugar content of GF and standard products, the small amount of sugar consumed per portion is unlikely to impact on consumer health. 

Wild et al. reported lower intakes of micronutrients in CD patients when compared to the standard population, particularly iron, folate, and magnesium [15], although Kinsey et al. concluded adequate intakes of iron in the coeliac diet [14]. The study found that 47% of patients had been prescribed a calcium and vitamin D supplement. On average, patients who had not been prescribed with a calcium supplement consumed less than the RNI. Low calcium intakes are supported by the findings of Kinsey et al. who observed intakes of 866 mg per day [14]. This is below the specific coeliac RNI of 1000 mg per day, which is advised as an osteoporosis prevention measure. Further research into the micronutrient intake of CD patients is needed. 

While bread and pasta are staples and major contributors to nutrient intake, other sources of nutrients need to be considered to assess nutrient intake in the CD population.

### 4.3. Fortification

GF products are currently excluded from fortification legislation, possibly because when the fortification legislation came into place, CD was relatively unknown. The Department for Environment Food & Rural Affairs [9] concluded in their review of the regulations that fortification is cheap, nutritionally beneficial for the population and convenient for manufacturers. The present study showed that fortification of bread contributes to nutrient intake, particularly of calcium (23.5% of RNI) and thiamin (24–30% of RNI). We propose that the mandatory fortification of GF foods is necessary to provide equivalent nutrition to the healthy population. Fortification levels for GF foods must be carefully considered, taking into account CD pathophysiology, including potential accumulation of metals [18].

### 4.4. Limitations

This study represents a survey of GF bread and pasta products available in the four major British supermarkets, through online retailing. While this represents over 70% of the market penetration in the UK, we did not survey the remaining 30% of retailing places, including specialist shops selling GF foods. The survey only revealed 49 GF products (bread and pasta) available. While this is a small sample which limits the generalizability of the results, it does reflect the limited availability of GF products. We had to exclude some bread categories (such as crumpets, muffins, naan bread) because the GF version of these products could not be found in these supermarkets. The nutrient analysis was based on back-of-pack information. While this is an improvement compared to studies using front-of-pack information, chemical analysis of food should be applied to verify nutrient content, especially micronutrient content. 

## 5. Conclusions

Despite improvements in the formulation and availability of GF in the last decade, GF foods are still less available and more expensive than gluten-containing versions. The macronutrient profile suggests that GF foods generally have adequate levels of fiber and sugar, but lower levels of protein and higher levels of fat, compared to their equivalent gluten-containing products. Very few GF foods were found to be fortified with micronutrients. As mandatory fortification of wheat flour is considered a successful health initiative for the general population, fortification should be extended to include GF products. Fortification of these products may reduce the risk of micronutrient deficiency and therefore deficiency-related diseases in CD patients.

## Figures and Tables

**Table 1:** Macronutrient and salt content of standard and gluten-free (GF) products per portion (100 g) and pasta (75 g) and the percentage contribution to reference nutrient intake (RNI).

Nutrition Attribute	RNI Value Per Day	Category	Standard Products		GF Products	
			Intake Per Portion	% Contribution to RNI	Intake Per Portion	% Contribution to RNI
Energy (kJ)	8400	White bread	1043	12	1016	12
		Brown bread	984	12	1035	12
		Seeded bread	1168	14	1113	13
		Pasta	1134	14	1117	13
Fat (g)	<70	White bread	3	4	5	7
		Brown bread	3	4	6	9
		Seeded bread	7	10	9	13
		Pasta	1	2	1	2
Saturates (g)	<20	White bread	1	3	0	2
		Brown bread	1	3	1	3
		Seeded bread	1	5	1	4
		Pasta	0	1	0	2
Carbohydrate (g)	260	White bread	46	18	42	16
		Brown bread	39	15	39	15
		Seeded bread	39	15	37	14
		Pasta	54	21	51	20
Sugars (g)	90	White bread	3	4	3	3
		Brown bread	3	4	2	2
		Seeded bread	4	4	2	3
		Pasta	2	2	0	0
Protein (g)	50	White bread	9	17	4	9
		Brown bread	10	21	8	16
		Seeded bread	11	22	6	12
		Pasta	9	18	5	9
Salt (g)	6	White bread	1	16	1	16
		Brown bread	1	15	1	15
		Seeded bread	1	15	1	15
		Pasta	0	1	0	1
Fiber (g)	30	White bread	3	9	7	22
		Brown bread	6	21	8	26
		Seeded bread	6	22	8	26
		Pasta	2	8	1	4

**Table 2:** Reference nutrient intake (RNI) for calcium, iron, niacin and thiamin and the contribution of 100 g of standard bread.

Micronutrient	RNI (mg/day)		Contribution to RNI (%)	
	Male	Female	Male	Female
Calcium	1000	1000	23.5	23.5
Iron	8.7	14.8	19	11
Niacin	18	14	9	11
Thiamin	1	0.8	24	30

## Supplementary Materials

The following are available online at http://www.mdpi.com/2072-6643/10/10/1370/s1. Table S1: List of products identified in the survey with their back-of-pack nutrient information (energy, macronutrients, and salt values in g per 100 g) and fortification; Table S2: Ingredient list for standard bread and pasta products identified in the supermarket survey; Table S3: Ingredient list for gluten-free (GF) bread and pasta products identified in the supermarket survey.
