# Supplementary material for: The Availability and Nutritional Adequacy of Gluten-Free Bread and Pasta
Source: Nutrients. 2018 Sep 25;10(10):1370. doi: 10.3390/nu10101370 (PMC6213709; doi:10.3390/nu10101370)
Supplement: Supplementary file 1 [file nutrients-10-01370-s001.zip › nutrients-354100-supplementary/Supplementary table 1.pdf]

Table 1. List of products identified in the survey with their back of pack nutrient information (energy, macronutrients and salt values in g per 100 grams) and fortification.

	Type	Product	Cost (£/100 g)	Energy kJ	Energy kcal	Fat	Of which saturates	Carbohy-drate	Of which Sugars	Fibre	Protein	Salt	Fortified with minerals and vitamins	Fortified with minerals only
White Bread	Standard	Hovis Soft White Medium Bread 800G	0.13	987	233	1.7	0.3	44.6	3.5	2.4	8.7	0.9	x	
		Warburtons Toastie Sliced White Bread 800G	0.13	1025	244	2	0.5	46.4	3	2.3	9.1	0.98	x	
		Tesco White Medium Bread 800G	0.06	1007	238	2.2	0.4	44.6	2.8	2.9	8.5	1	x	
		Kingsmill Soft White Medium 800g	0.11	1007	238	2	0.6	45.6	3.8	2.7	8	1	x	
		Tesco Everyday Value Sliced White Bread 800G	0.05	991	234	1.5	0.3	46.3	4.9	2.6	7.5	1	x	
		Warburtons Farmhouse White Bread 800G	0.17	1021	243	2.5	0.5	45	2.2	2.3	9	0.98	x	
		Sainsbury's Soft Medium Sliced White Bread 800g	0.07	1013	239	2.2	0.5	45.6	2.5	2.3	8.1	0.89	x	
		Sainsbury's Medium Sliced White Bread, Basics 800g	0.05	1005	238	1.9	0.3	45.5	4.1	3.2	8.1	0.9	x	
		Warburtons Toastie Thick Sliced White Bread 400g	0.13	1025	244	2	0.5	45.4	2.9	2.1	9.1	0.98	x	
		Sainsbury's White Farmhouse Bread 800g	0.13	1027	242	0.9	0.1	48.1	3.2	2.7	9.1	0.93	x	
		ASDA Baker's Selection Square Cut Medium White Bread	0.74	1013	239	2.2	0.5	46	2.5	2.3	8.1	0.89	x	
		ASDA Extra Special Farmhouse White Bread	0.11	1006	238	2	0.3	45	3.8	2.2	8.8	0.88	x	
		Roberts Bakery White Bloomer 600g	0.19	1039	246	3.2	0.6	43.7	2.9	3.8	8.6	1	x	
		Morrisons Medium White Loaf 800g	0.06	985	232	1.2	0.2	45	2.8	2.6	9.1	0.9	x	
		M savers White Loaf 720g	0.05	1068	252	1.1	0.2	50.4	4.1	3.2	8.5	0.9	x	
		Warburtons Sliced White Rolls 8 per pack	0.27	1113	265	3.9	0.8	46.6	3	2.4	9.7	0.98	x	
		ASDA Baker's Selection Super Soft White Rolls	0.31	1151	272	4.6	0.7	49	3.4	2.5	7.5	0.93	x	
		Kingsmill Soft White Sliced Rolls	0.23	1138	270	5.6	1.1	45.5	3.5	2.7	8	0.85	x	
		Tesco White Baps 4 Pack	0.13	1093	259	3.9	2	44	3.7	3	10.4	0.8	x	
		Kingsmill Soft White Rolls 6 Pack	0.27	1138	270	4.5	0.9	47.9	4.6	2.7	8	1	x	
		Sainsbury's White Jumbo Hot Dog Rolls x6	0.19	1058	250	3.3	0.9	45	4.3	3.5	8.4	0.98	x	
	GF	Tesco Free From White Rolls 4 Pack	0.61	835	199	4.1	0.3	30.9	1.5	8.9	5.1	1.1		
		Tesco Free From Sliced White Bread 550G	0.38	844	201	4.1	0.3	32.5	0.6	6.4	5.2	1		
		Schar White Ciabatta Rolls 200G	1	900	213	1.8	0.3	41	2.7	8.3	4.1	1		
		Ds Wholesome White Sliced Loaf 300G	0.83	1009	239	3.4	0.7	45	3.3	7.3	3.5	1		
		Genius Gluten Free Toastie 500G	0.54	1142	272	6.1	0.4	47	3.9	9.3	2.4	0.9	x	
		Newburn Bakehouse Mini Loaf White 300G	0.58	1046	249	6.5	0.6	37.9	1.6	5.8	6.7	0.83		
		Warburtons Newburn Bakehouse White Sourdough Artisan	0.8	1040	248	5.9	0.6	39.1	2.6	5.4	6.8	0.84		
		ASDA Free From White Sliced Loaf	0.37	1126	268	7.2	0.5	46	0.9	5.4	2.3	0.92		
		Schar Gluten Free Wholesome White Loaf	0.78	1009	239	3.4	0.7	45	3.3	7.3	3.5	1		

		Genius Gluten Free White Rolls	0.81	1176	280	7	0.5	49.2	6.9	5.9	2	0.67		
		Genius Gluten Free Soft White Sandwich Loaf	0.46	1143	272	6.1	0.4	47	3.9	9.3	2.4	0.9	x	
		Schar Gluten Free Hamburger Buns	0.95	855	202	1.1	0.1	42	7.7	5.7	3.2	0.87		
		Schar Gluten Free Panini Rolls	0.88	1019	241	3	0.5	46	2.7	7.4	3.9	1.1		
		Warburtons Gluten Free White Farmhouse Loaf 560g	0.54	983	234	5.4	0.5	35.8	1.3	5.7	7.8	0.8		x
		Warburtons Gluten Free White Rolls 4pk	0.96	1025	244	6.2	0.5	37.1	1.5	5.4	7.3	0.9		x
		Warburtons Tiger Artisan Bloomer, Gluten Free 400g	0.88	966	230	5.3	0.5	38.5	2.6	5.5	7.1	0.84		
		Schar Gluten Free White Rolls 348g	0.69	1036	245	3.6	0.5	47	0.8	5.5	3.5	1.3		
		Morrisons Free From Gluten Free White Bread 350g	0.48	1061	252	6.4	0.5	43.6	1.5	6.1	2	0.86		
		Morrisons Free From Gluten Free 4 White Rolls 4 x 65g	0.65	1091	259	6.6	0.5	44.9	1.6	5.6	2.3	0.86		
Brown Bread	Standard	Tesco Wholemeal Medium Bread 800G	0.06	991	235	3	0.5	36.9	3.5	7.1	11.5	1		
		Hovis Wholemeal Medium Bread 800G	0.13	934	221	1.8	0.4	37.8	4.1	6.8	10	0.9	x	
		Kingsmill Tasty Wholemeal Medium Bread 800G	0.11	984	233	2.8	0.6	38.6	4.3	6.3	10.2	1		
		Warburtons Wholemeal Medium Bread 800G	0.13	974	231	2.8	0.5	37.8	2.4	6.4	10.6	0.95		
		Tesco Everyday Value Medium Sliced Wholemeal Bread 800G	0.05	975	231	2.5	0.4	39	3.4	7	9.6	0.9		
		Allinson's Classic Wholemeal Loaf 650G	0.19	974	231	2.6	0.4	38.7	2.8	6.7	9.8	1		
		Sainsbury's Medium Sliced Wholemeal Bread 800g	0.07	986	234	2.9	0.6	36.7	3	6.6	11.9	0.88		
		Sainsbury's Thick Sliced Wholemeal Bread, SO Organic 800g	0.18	1007	239	3.2	0.6	39.3	2.7	7.2	9.6	0.88		
		ASDA Baker's Selection Square Cut Medium Wholemeal Bread	0.07	986	234	2.9	0.6	37	3	6.6	12	0.88		
		Morrisons Wholemeal Medium Loaf 800g	0.06	913	216	1.4	0.3	38	2.8	6.5	9.6	0.89		
		Morrisons Wholemeal Toastie Loaf 800	0.06	913	216	1.4	0.3	38	2.8	6.5	9.6	0.89		
		Morrisons Wholemeal Rolls 6 per pack	0.13	1006	238	2.8	0.4	40	3.2	5.9	10.3	0.8		
		Kingsmill Tasty Wholemeal Rolls 6 Pack	0.25	1032	245	3.7	0.9	39.1	4.7	6.5	10.5	0.93		
		Warburtons Brown Sandwich Thins 6 Pack	0.42	1058	252	2.8	0.9	45.2	3.9	3.5	9.9	0.98	x	
		Tesco Wholemeal Batch Rolls 6 Pack	0.11	1020	242	3.3	1.3	39.8	5	5.6	10.4	0.7	x	
	GF	Genius Brown Sliced Bread 535G	0.51	1131	269	6.6	0.5	45	2.4	10	2.5	0.9	x	
		Schar Brown Ciabatta Rolls 200G	1	1150	274	8.1	1	40	3.7	8.9	5.8	1		
		Tesco Free From Brown Bread 550G	0.38	811	193	4	0.3	29.3	0.7	9.3	5.3	1		
		Newburn Bakehouse Brown Loaf 560g	0.53	983	234	5.4	0.5	35.8	1.3	5.7	7.8	0.8		x
		ASDA Free From Brown Sliced Loaf	0.37	1098	261	7	0.5	44	1.6	5.8	2.2	0.85		
Seeded Bread	Standard	Hovis Seed Sensations Seven Seeds Medium Sliced Seeded Bread 800g	0.16	1171	278	6.6	0.6	42	3.8	5.3	10	0.88	x	
		Warburtons Thick Sliced Seeded Bread 800g	0.19	1231	293	8.9	1.3	39.7	2.6	6	10.6	0.95	x	

		Hovis Seed Sensations Soft Granary Medium Sliced Seeded Bread 800g	0.19	1157	275	6.6	0.8	41.3	4.6	4.9	10.1	0.83	x	
		Sainsbury's Multiseeded Medium Sliced Bread Half Bloomer, Taste the Difference 400g	0.13	1079	256	3.6	0.4	39.7	4.3	8.8	12	0.84	x	
		Kingsmill Medium Sliced Seeded Bread Half Loaf 470g	0.17	1033	245	4.8	1.2	34.4	3.6	7.9	12.2	0.8		
		Hovis Seed Sensations Sunflower & Pumpkin Medium Sliced Seeded Bread 400g	0.25	1260	301	10.8	1.6	32.4	2.8	9	14	0.96	x	
		Allinson's Seeds & Grains Medium Sliced Bread 650g	0.15	1270	303	10.9	1.7	38.1	3.2	4.9	10.6	0.9	x	
		Sainsbury's Ancient Grains Medium Sliced White Bread, Taste the Difference 800g	0.13	1113	264	3.6	0.7	44.1	4.7	5.4	11	0.88	x	
		Tesco Multiseed Farmhouse Batch Bread 800G	0.11	1127	268	6.5	0.7	39.9	3.2	6	9.4	1	x	
		Tesco Finest Super Seeded Bread 800G	0.14	1203	287	8.9	1.1	37.8	3.4	6.6	10.5	1	x	
		Tesco Finest Sunflower And Pumpkin Cob Bread 600G	0.18	1255	299	10.9	1.7	34.7	3.4	6.6	12.3	0.9	x	
		Morrisons The Best Seeded Loaf 800g	0.1	1144	272	6.4	0.7	39.6	2.3	5.2	11.3	0.67	x	
		Burgen Soya & Linseed Loaf 800g	0.18	1201	287	11	1.5	26.9	4.2	9.8	15.2	0.75	x	
		Warburtons Malted Grain & Seeds 400g	0.3	1170	276	4.6	0.8	43.4	3.7	5.4	12.6	1	x	
		Roberts Bakery Seeded Bloomer 600g	0.2	1154	275	8.2	1.1	35.4	2.8	8.7	10.6	1	x	
		Tesco Multiseed Deli Rolls 4 Pack	0.4	1192	283	4.5	1.2	47.5	4.9	5.8	10.1	0.9	x	
		Morrisons Medium Granary Rolls 4 per pack	0.28	1051	249	2.7	0.6	43.5	3.1	5.4	9.9	0.81	x	
		Tesco Seeded Burger Buns	0.11	1097	260	2.9	1.3	46.7	2.8	4.1	9.6	0.8	x	
		Sainsbury's Large Multi Seeded Deli Rolls x4	0.34	1210	288	8.3	1.1	40	4.9	7.1	9.9	0.89	x	
		Sainsbury's Wholemeal Seeded Rolls, SO Organic x4	0.34	1234	294	10.3	1.1	36	2.7	6.3	11.2	0.85	x	
	GF	Genius Gluten Free Triple Seeded Farmhouse Bread 535g	0.52	1194	285	9.4	0.8	42.5	3.4	7.8	3.7	0.9	x	
		Schar Wholesome Seeded Sliced Bread 300G	0.83	1042	248	6.2	0.9	40	2.8	7.4	4.3	0.85		
		Tesco Free From Sliced Seeded Bread 550G	0.38	951	227	8.8	0.8	25.7	0.5	8.7	7	1		
		Genius Gluten Free Wholesome Five Seeded 535G	0.52	1194	285	9.4	0.8	42.3	3.4	7.8	3.7	0.9		
		Tesco Free From Multiseed Slice Bread 400G	0.45	1268	302	8.5	0.8	49.5	1.1	6.6	3.5	0.7		
		Schar Wholesome Vitality Loaf Gluten Free 350G	0.74	1099	262	9.2	1.1	36	0.9	8.8	4.5	1		
		Tesco Free From Brown Seeded Rolls 4 Pack	0.61	931	222	7.3	0.6	28.8	0.4	7.4	6.7	1		
		Genius Multiseed Rolls 4 Pack	0.71	1252	299	11.2	1	40.6	5.7	10	3.9	0.8	x	
		Newburn Bakehouse Mini Loaf Seeded 300G	0.58	1163	277	10.9	1	33.5	1.5	7	7.7	0.8		
		Burgen Free From Sunflower And Chia Seed Bread 500G	0.55	1201	286	10.3	0.8	39.7	2.7	5.4	6	1		
		Bfree Brown Seeded Bloomer High Fibre 400G	0.75	928	222	5	0.6	32	0.5	9.7	7.8	1		
		Schar Seeded Ciabatta Gluten Free 210G	0.98	1241	296	11	1.3	38	2.3	8	7.3	0.75		
		Tesco Free From Ancient Grain Cob 400G	0.75	927	221	6.1	0.7	30.3	1.8	8	7.2	1		

		Kelkin Sourdough Multiseed 200G	1	1240	296	10	1.2	38	8.9	11	7.1	1.1		
		Morrisons Free From Gluten Free Seeded Bread 350g	0.48	1072	255	7.9	0.7	39.9	1.3	6.4	3	0.75		
		Warburtons Seeded Farmhouse Loaf Gluten Free 560g	0.54	1100	262	9.1	0.8	33	1.2	6.5	8.7	0.8		
Pasta	Standard Dry Values	Tesco Fusilli Pasta Twists 1Kg	0.09	1526	360	1.4	0.3	73	2.4	2.6	12.5	0.1		
		Tesco Everyday Value Penne 500G	0.06	1490	351	1.5	0.5	72	2.5	0.8	12	0.1		
		Napolina Short Spaghetti 500G	0.25	1508	356	1.5	0.3	72	3	3	12	0.01		
		De Cecco Linguine 500G	0.31	1493	352	1.5	0.3	70.2	3.4	2.9	13	0.01		
		Barilla Spaghetti 500G	0.2	1521	359	2	0.5	71.2	3.5	3	12.5	0.013		
		Tesco Pasta Animals 500G	0.18	1450	343	1.4	0.3	68	2.8	8	10.5	0.1		
		Tesco Margheritine Soup Pasta 250G	0.2	1526	360	1.4	0.3	73	2.4	2.6	12.5	0.1		
		De Cecco Fusilli 500G	0.31	1493	352	1.5	0.3	70.2	3.4	2.9	13	0.01		
		Barilla Fusilli Pasta 500g	0.25	1521	359	2	0.5	71.7	3.5	3	12	0.01		
		Morrisons Conchiglie 500g	0.12	1581	373.33	2.00	0.53	74.93	2.93	2.40	6.53	0		
		Tesco Spaghetti 2Kg	0.09	1526	360	1.4	0.3	73	2.4	2.6	12.5	0.1		
	GF Dry Values	Sainsbury's Deliciously Freefrom Fusilli 500g	0.26	1439	340	2.4	0.8	72	0.6	1.8	6.6	0.01		
		Doves Farm Gluten Free Organic Penne Pasta 500g	0.42	1516	357	1.3	0.4	78	0.2	1.4	7.4	0		
		Sainsbury's Buckwheat Penne 500g	0.43	1555	368	3.2	0.4	34.5	0.2	3	6.4	0		
		Sainsbury's Deliciously Freefrom Spaghetti 500g	0.26	1439	340	2.4	0.4	36	0.5	0.9	3.3	0.01		
		Tesco Free From Fusilli Pasta 500G	0.24	1505	355	1	0.2	78.4	0.5	2	7	0.1		
		Barilla Gluten Free Spaghetti 400G	0.31	1524	359	1.8	0.3	78.7	1.2	1.1	6.5	0.01		
		Tesco Free From Penne Pasta 500G	0.25	1505	355	1	0.2	78.4	0.5	2	7	0.1		
		Tesco Free From Lasagne Sheets 250G	0.5	1505	355	1	0.2	78.4	0.5	2	7	0.1		
		Sainsbury's Deliciously Freefrom Penne 500g	0.26	1439	340	2.4	0.8	72	0.6	1.8	6.6	0.01		
		Morrisons Free From Fusilli 500g	0.27	1463	344	1	0	77	0.3	1.9	3.7	0.12		
