# Supplementary material for: The Availability and Nutritional Adequacy of Gluten-Free Bread and Pasta
Source: Nutrients. 2018 Sep 25;10(10):1370. doi: 10.3390/nu10101370 (PMC6213709; doi:10.3390/nu10101370)
Supplement: Supplementary file 1 [file nutrients-10-01370-s001.zip › nutrients-354100-supplementary/Supplementary table 2.pdf]

Supplementary Table 2: Ingredient list for standard bread and pasta products identified in the supermarket survey.

	Product	Ingredients
White	Hovis Soft White Medium Bread 800G	Wheat Flour (with added Calcium, Iron, Niacin, Thiamin), Water, Yeast, Soya Flour, Salt, Preservative: E282, Emulsifiers: E471, E472e, Flour Treatment Agent: Ascorbic Acid
	Warburtons Toastie Sliced White Bread 800G	Wheat Flour [with Calcium, Iron, Niacin (B3) and Thiamin (B1)], Water, Yeast, Salt, Vegetable Oil (Rapeseed, Sustainable Palm), Soya Flour, Preservative: Calcium Propionate (added to inhibit mould growth), Emulsifiers: E481, E472e, Flour Treatment Agent: Ascorbic Acid (Vitamin C)
	Tesco White Medium Bread 800G	Wheat Flour (Wheat Flour, Calcium Carbonate, Iron, Niacin, Thiamin), Water, Yeast, Salt, Rapeseed Oil, Spirit Vinegar, Emulsifiers (Mono- and Di-Acetyltartaric Esters of Mono- and Di-Glycerides of Fatty Acids, Sodium Stearoyl-2-Lactylate, Mono- and Di-Glycerides of Fatty Acids), Soya Flour, Preservative (Calcium Propionate), Palm Oil, Flour Treatment Agent (Ascorbic Acid)
	Kingsmill Soft White Medium 800g	Wheat Flour (with Calcium, Iron, Niacin (B3) and Thiamin (B1)), Water, Yeast, Salt, Soya Flour, Vegetable Oils (Rapeseed, Sustainable Palm), Emulsifiers: E471, E472e, E481, Preservative: Calcium Propionate (added to inhibit mould growth), Vinegar, Flour Treatment Agent: Ascorbic Acid (Vitamin C)
	Tesco Everyday Value Sliced White Bread 800G	Wheat Flour (Wheat Flour, Calcium Carbonate, Iron, Niacin, Thiamin), Water, Yeast, Salt, Rapeseed Oil, Soya Flour, Spirit Vinegar, Preservative (Calcium Propionate), Emulsifier (Mono- and Di-Acetyltartaric Esters of Mono- and Di-Glycerides of Fatty Acids), Palm Oil, Flour Treatment Agent (Ascorbic Acid)
	Warburtons Farmhouse White Bread 800G	Wheat Flour [with Calcium, Iron, Niacin (B3) and Thiamin (B1)], Water, Yeast, Vegetable Oil (Rapeseed, Sustainable Palm), Salt, Flavouring, Soya Flour, Preservative: Calcium Propionate (added to inhibit mould growth), Emulsifiers: E471, E481, Flour Treatment Agents: Ascorbic Acid (Vitamin C), E920 (Vegetarian)
	Sainsbury's Soft Medium Sliced White Bread 800g	Fortified Wheat Flour (Wheat Flour, Calcium Carbonate, Iron, Niacin, Thiamin), Water, Yeast, Salt, Rapeseed Oil, Spirit Vinegar, Emulsifiers:Mono- and Diacetyl Tartaric Acid Esters of Mono- and Diglycerides of Fatty Acids, Sodium Stearoyl-2-Lactylate, Mono- and Diglycerides of Fatty Acids; Soya Flour, Preservative:Calcium Propionate; Palm Oil, Flour Treatment Agent:Ascorbic Acid .
	Sainsbury's Medium Sliced White Bread, Basics 800g	FortifiedBritish Wheat Flour (Wheat Flour, Calcium Carbonate, Iron, Niacin,Thiamin), Water, Yeast, Salt, Spirit Vinegar, Soya Flour, Emulsifier: Mono- and Diacetyl Tartaric Acid Esters of Mono- and Diglycerides of Fatty Acids; Rapeseed Oil, Flour Treatment Agent: Ascorbic Acid; Palm Oil.
	Warburtons Toastie Thick Sliced White Bread 400g	Wheat Flour [with Calcium, Iron, Niacin (B3) and Thiamin (B1)], Water, Yeast, Salt, Vegetable Oil (Rapeseed, Sustainable Palm), Soya Flour, Preservative: Calcium Propionate (added to inhibit mould growth), Emulsifiers: E481, E472e, Flour Treatment Agent: Ascorbic Acid (Vitamin C)
	Sainsbury's White Farmhouse Bread 800g	Fortified Wheat Flour (Wheat Flour, Calcium Carbonate, Iron, Niacin, Thiamin), Water, Rapeseed Oil, Yeast, Salt, Wheat Gluten, Soya Flour, Flour Treatment Agent:Ascorbic Acid;, Processing Aid:Enzymes;.
	ASDA Baker's Selection Square Cut Medium White Bread	Fortified Wheat Flour [Wheat Flour, Calcium Carbonate, Iron, Niacin (B3), Thiamin (B1)] , Water , Yeast , Salt , Vegetable Oils [Rapeseed Oil, Palm Oil] , Spirit Vinegar , Emulsifiers (Mono- and Diacetyl Tartaric Acid Esters of Mono- and Diglycerides of Fatty Acids, Sodium Stearoyl-2-Lactylate, Mono- and Diglycerides of Fatty Acids) , Soya Flour , Preservative (Calcium Propionate) , Flour Treatment Agent (Ascorbic Acid) .

	ASDA Extra Special Farmhouse White Bread	Fortified Wheat Flour [Wheat Flour, Calcium Carbonate, Iron, Niacin (B3), Thiamin (B1)] , Water , Sourdough (7.2%) [Wheat and Rye Sourdough, Spirit Vinegar, Salt, Wheat Starch] , Yeast , Fermented Wheat Flour , Soya Flour , Wheat Protein , Vegetable Oils [Rapeseed Oil, Palm Oil] , Salt , Spirit Vinegar , Dried Wheat Sourdough , Flour Treatment Agent (Ascorbic Acid) .
	Roberts Bakery White Bloomer 600g	Wheat Flour (with added Calcium, Iron, Niacin and Thiamine), Water, Wheat Sourdough (Wheat Flour, Water, Starter Culture, Vinegar, Salt, Wheat Starch), Yeast, Olive Oil (1.5%), Durum Wheat Semolina (0.8%), Salt, Emulsifiers: E471, E472e, E481 (from Sunflower and Palm), Soya Flour, Preservative: Calcium Propionate (to Inhibit Mould Growth), Rapeseed Oil, Flour Treatment Agent: Ascorbic Acid (Vitamin C)
	Morrisons Medium White Loaf 800g	Fortified Wheat Flour (Wheat Flour, Iron, Thiamin, Nicotinic Acid, Calcium Carbonate), Water, Salt, Yeast, Soya Flour, Preservative (Calcium Propionate), Emulsifier (Mono- and Diacetyltartaric Acid Esters of Mono- and Diglycerides of Fatty Acids), Flour Treatment Agent (Ascorbic Acid), Rapeseed Oil, Folic Acid
	M savers White Loaf 720g	Fortified Wheat Flour (Wheat Flour, Iron, Thiamin, Nicotinic Acid, Calcium Carbonate), Water, Salt, Soya Flour, Emulsifiers (Mono- and Diacetyl Tartaric Acid Esters of Mono- and Diglycerides of Fatty Acids), Preservative (Calcium Propionate), Yeast, Flour Treatment Agent (Ascorbic Acid), Folic Acid
	Warburtons Sliced White Rolls 8 per pack	Wheat Flour [with Calcium, Iron, Niacin (B3) and Thiamin (B1)] , Water , Vegetable Oil (Rapeseed, Sustainable Palm) , Yeast , Salt , Sugar , Emulsifiers: E472e, E471, E481 , Soya Flour , Gelling Agent: E466 , Preservative: Calcium Propionate (added to inhibit mould growth) , Flour Treatment Agents: Ascorbic Acid (Vitamin C), E920 (Vegetarian) .
	ASDA Baker's Selection Super Soft White Rolls	Fortified Wheat Flour [Wheat Flour, Calcium Carbonate, Iron, Niacin (B3), Thiamin (B1)] , Water , Maize Flour , Rapeseed Oil , Sugar , Salt , Emulsifiers (Mono- and Diglycerides of Fatty Acids, Mono- and Diacetyl Tartaric Acid Esters of Mono and Duglycerides of Fatty Acids, Sodium Stearoyl-2-Lactylate) , Yeast , Spirit Vinegar , Palm Oil , Soya Flour , Wheat Starch , Flour Treatment Agent (Ascorbic Acid) .
	Kingsmill Soft White Sliced Rolls	Wheat Flour (with Calcium, Iron, Niacin (B3), Thiamin (B1)) , Water , Vegetable Oils (Rapeseed, Sustainable Palm) , Yeast , Sugar , Salt , Soya Flour , Emulsifiers: E471, E472e , Vinegar , Preservative: Calcium Propionate (added to inhibit mould growth) , Flour Treatment Agent: Ascorbic Acid (Vitamin C
	Tesco White Baps 4 Pack	Wheat Flour (Wheat Flour, Calcium Carbonate, Iron, Niacin, Thiamin), Water, Palm Oil, Dextrose, Yeast, Salt, Emulsifiers (Mono- and Di-Glycerides of Fatty Acids, Mono- and Di-Acetyltartaric Esters of Mono- and Di-Glycerides of Fatty Acids), Spirit Vinegar, Fermented Wheat Flour, Soya Flour, Rapeseed Oil, Flour Treatment Agents (Ascorbic Acid, L-Cysteine Hydrochloride), Wheat Starch
	Kingsmill Soft White Rolls 6 Pack	Wheat Flour (with Calcium, Iron, Niacin (B3) and Thiamin (B1)), Water, Vegetable Oils (Rapeseed, Sustainable Palm), Yeast, Sugar, Salt, Soya Flour, Emulsifiers: E471, E472e, Vinegar, Preservative: Calcium Propionate (added to inhibit mould growth), Flour Treatment Agent: Ascorbic Acid (Vitamin C)
	Sainsbury's White Jumbo Hot Dog Rolls x6	Fortified British Wheat Flour (Wheat Flour, Wheat Gluten, Calcium Carbonate, Iron, Niacin, Thiamin), Water, Rapeseed Oil, Yeast, Sugar, Salt, Spirit Vinegar, Palm Oil, Fermented Wheat Flour, Soya Flour, Palm Fat, Flour Treatment Agent:Ascorbic Acid , Wheat Starch.
Brown	Tesco Wholemeal Medium Bread 800G	Wholemeal Wheat Flour, Water, Wheat Gluten, Yeast, Salt, Rapeseed Oil, Spirit Vinegar, Soya Flour, Caramelised Sugar, Emulsifier (Mono- and Di-Acetyltartaric Esters of Mono- and Di-Glycerides of Fatty Acids), Preservative (Calcium Propionate), Palm Oil, Flour Treatment Agent (Ascorbic Acid)
Brown	Hovis Wholemeal Medium Bread 800G	Wholemeal Flour (Wheat), Water, Caramelised Sugar, Yeast, Wheat Protein, Soya Flour, Salt, Wheat Flour (with added Calcium, Iron, Niacin, Thiamin), Emulsifiers: E471, E472e, E481, Preservative: E282, Vegetable Fat (Palm, Rapeseed), Flour Treatment Agent: Ascorbic Acid, This product contains 62% wholegrains from Wholemeal Flour

	Kingsmill Tasty Wholemeal Medium Bread 800G	Wholemeal Wheat Flour, Water, Kibbled Malted Wheat (4%), Sugar, Wheat Protein, Yeast , Salt, Vegetable Oils (Rapeseed, Sustainable Palm), Malted Barley Flour, Emulsifiers: E471, E472e, Vinegar, Soya Flour, Preservative: Calcium Propionate (added to inhibit mould growth), Flour Treatment Agent: Ascorbic Acid (Vitamin C), With 62% Whole grain (Wholemeal Wheat Flour, Kibbled Malted Wheat, Malted Barley Flour)
	Warburtons Wholemeal Medium Bread 800G	Wholemeal Wheat Flour (59%), Water, Yeast, Salt, Vegetable Oil (Rapeseed, Sustainable Palm), Wheat Gluten, Emulsifiers: E471, E481, E472e, Soya Flour, Preservative: Calcium Propionate (added to inhibit mould growth), Flour Treatment Agent: Ascorbic Acid (Vitamin C)
	Tesco Everyday Value Medium Sliced Wholemeal Bread 800G	Wholemeal Wheat Flour, Water, Yeast, Salt, Spirit Vinegar, Wheat Protein, Rapeseed Oil, Soya Flour, Emulsifier (Mono- and Di-Acetyltartaric Esters of Mono- and Di-Glycerides of Fatty Acids), Preservative (Calcium Propionate), Palm Oil, Flour Treatment Agent (Ascorbic Acid)
	Morrisons Wholemeal Medium Loaf 800g	Wholemeal Wheat Flour, Water, Salt, Yeast, Preservative (Calcium Propionate), Soya Flour, Emulsifier (Mono- and Diacetyltartaric Acid Esters of Mono- and Diglycerides of Fatty Acids), Wheat Flour, Flour Treatment (Ascorbic Acid), Folic Acid
	Morrisons Wholemeal Toastie Loaf 800	Wholemeal Wheat Flour, Water, Salt, Yeast, Soya Flour, Preservative (Calcium Propionate), Emulsifiers (Mono- and Diacetyltartaric Acid Esters of Mono- and Diglycerides of Fatty Acids), Wheat Flour, Flour Treatment Agent (Ascorbic Acid), Folic Acid
	Kingsmill Tasty Wholemeal Rolls 6 Pack	Wholemeal Wheat Flour, Water, Kibbled Malted Wheat (4%), Wheat Semolina, Sugar, Wheat Protein, Vegetable Oils (Rapeseed, Sustainable Palm), Yeast, Salt, Malted Barley Flour, Emulsifiers: E471, E472e, Soya Flour, Vinegar, Preservative: Calcium Propionate (added to inhibit mould growth), Flour Treatment Agent: Ascorbic Acid (Vitamin C), With 56% Whole Grain (Wholemeal Wheat Flour, Kibbled Malted Wheat, Malted Barley Flour)
	Warburtons Brown Sandwich Thins 6 Pack	Wheat Flour [with Calcium, Iron, Niacin (B3) and Thiamin (B1)], Wholemeal Wheat Flour, Water, Vegetable Oil (Rapeseed, Sustainable Palm), Wheat Gluten, Dextrose, Salt, Yeast, Barley Malt Flavour, Sugar, Stabiliser: Xanthan Gum, Emulsifiers: E471, E481, Gelling Agent: E466, Caramelised Sugar, Soya Flour, Preservative: Calcium Propionate (added to inhibit mould growth), Flour Treatment Agents: Ascorbic Acid (Vitamin C), E920 (Vegetarian)
	Tesco Wholemeal Batch Rolls 6 Pack	Wholemeal Wheat Flour, Water, Wheat Flour (Wheat Flour, Calcium Carbonate, Iron, Niacin, Thiamin), Palm Oil, Dextrose, Yeast, Salt, Spirit Vinegar, Soya Flour, Fermented Wheat Flour, Emulsifiers (Mono- and Di-Glycerides of Fatty Acids, Mono- and Di-Acetyltartaric Esters of Mono- and Di-Glycerides of Fatty Acids), Malted Barley, Flour Treatment Agents (Ascorbic Acid, L-Cysteine Hydrochloride), Wheat Starch
	Hovis Seed Sensations Seven Seeds Medium Sliced Seeded Bread 800g	Wheat Flour (with added Calcium, Iron, Niacin, Thiamin), Water, Seed Mix (13%) (contains: Toasted Brown Linseed, Toasted Sunflower Seeds, Pumpkin Seeds, Sunflower Seeds, Millet Seeds, Golden Linseed, Poppy Seeds), Yeast, Wheat Protein, Salt, Soya Flour, Caramelised Sugar, Malted Barley Flour, Barley Flour, Preservative: E282, Emulsifier: E472e, Vegetable Fat (Rapeseed, Palm), Barley Fibre, Flour Treatment Agent: Ascorbic Acid, Wheat Starch
	Warburtons Thick Sliced Seeded Bread 800g	Wheat Flour [with Calcium, Iron, Niacin (B3) and Thiamin (B1)], Water, Seed Mix (12%) (Sesame Seed, Sunflower Seed, Brown Linseed, Millet Seed, Poppy Seed), Yeast, Malted Barley Flour, Vegetable Oil (Rapeseed, Sustainable Palm), Wheat Gluten, Sugar, Salt, Soya Flour, Emulsifiers: E471, E481, E472e, Preservative: Calcium Propionate (added to inhibit mould growth), Flour Treatment Agent: Ascorbic Acid (Vitamin C)
Seeded	Hovis Seed Sensations Soft Granary Medium Sliced Seeded Bread 800g	Wheat Flour (with added Calcium, Iron, Niacin, Thiamin), Water, Seed Mix (10%) (contains: Toasted Brown Linseed, Toasted Sunflower Seeds, Millet Seeds, Pumpkin Seeds, Golden Linseed, Poppy Seeds), Grain Mix (3%) (contains: Granary Malted Wheat Flakes, Toasted Wheat, Toasted Rye), Wheat Protein, Yeast, Soya Flour, Salt, Caramelised Sugar, Malted Barley Flour, Barley Flour, Preservative: E282, Emulsifier: E472e, Vegetable Fat (Rapeseed, Palm), Barley Fibre, Flour Treatment Agent: Ascorbic Acid

	Sainsbury's Multiseeded Medium Sliced Bread Half Bloomer, Taste the Difference 400g	Fortified British Wheat Flour (Wheat Flour, Calcium Carbonate, Iron, Niacin, Thiamin), Water, Rye Flour, Sunflower Seeds (3.5%), Linseed (3.5%), Pearl Barley (2.5%), Yeast, Invert Sugar, Millet (1.7%), Malted Wheat Flakes, Pumpkin Seed (0.9%), Salt, Poppy Seed (0.7%), Rapeseed Oil, Soya Flour, Oats (0.4%), Fermented Wheat Flour, Malted Barley Flour, Malted Wheat, Malted Wheat Flour, Flour Treatment Agent: Ascorbic Acid.
	Kingsmill Medium Sliced Seeded Bread Half Loaf 470g	Wholemeal Wheat Flour, Water, Wheat Protein, Yeast, Malted Wheat Flakes, Toasted Kibbled Soya, Mixed Seeds (6%) (Sunflower Seeds, Golden Linseed, Brown Linseed, Poppy Seeds, Millet Seeds), Sugar, Malted Barley Flour, Salt, Vinegar, Soya Flour, Emulsifier: E472e, Vegetable Oils (Rapeseed, Sustainable Palm), Preservative: Calcium Propionate (added to inhibit mould growth), Flour Treatment Agent: Ascorbic Acid (Vitamin C)
	Hovis Seed Sensations Sunflower & Pumpkin Medium Sliced Seeded Bread 400g	Water, Wheat Flour (with added Calcium, Iron, Niacin, Thiamin), Wholemeal Flour (Wheat), Sunflower Seeds (8%), Toasted Sunflower Seeds (2%), Toasted Pumpkin Seeds (2%), Wheat Protein, Yeast, Pinhead Oats, Salt, Soya Flour, Pumpkin Seeds (0.5%), Oat Bran, Jumbo Oats, Barley Flour, Emulsifier: E472e, Wheat Bran, Preservative: E282, Vegetable Fat (Rapeseed, Palm), Molasses, Sugar, Barley Fibre, Malted Barley Flour, Flour Treatment Agent: Ascorbic Acid
	Allinson's Seeds & Grains Medium Sliced Bread 650g	Wheat Flour (with calcium, Iron, Niacin (B3), and Thiamin (B1)), Water, Mixed Seeds (16%) (Sunflower Seeds, Brown Linseed, Pumpkin Seeds, Golden Linseed, Poppy Seeds), Vegetable Oils (Rapeseed, Sustainable Palm), Millet (2%), Rye Flour (1.5%), Wheat Protein, Yeast, Salt, Fermented Wheat Flour, Malted Barley Flour, Vinegar, Emulsifiers: E471, E472e, E481, Soya Flour, Psyllium Fibre, Flour Treatment Agent: Ascorbic Acid (Vitamin C)
	Sainsbury's Ancient Grains Medium Sliced White Bread, Taste the Difference 800g	Fortified Wheat Flour (Wheat Flour, Calcium Carbonate, Iron, Niacin, Thiamin), Water, Sprouted Spelt (7%), Sprouted Naked Barley (6%), Yeast, Wheat Gluten, Millet (3%), Buckwheat (2.5%), Quinoa (2.5%), Dark Muscovado Sugar , Wholemeal Spelt Flour, Chia (Salvia Hispanica) Seeds, Teff Flour, Malted Barley Flour, Salt, Fermented Wheat Flour, Rapeseed Oil, Golden Linseed, Soya Flour, Spirit Vinegar, Palm Oil, Flour Treatment Agent:Ascorbic Acid .
	Tesco Multiseed Farmhouse Batch Bread 800G	Wheat Flour (Wheat Flour, Calcium Carbonate, Iron, Niacin, Thiamin), Water, Mixed Seeds (13%) (Linseed, Sunflower Seeds, Millet, Poppy Seeds, Pumpkin Seed), Wheat Gluten, Yeast, Salt, Barley Malt Flour, Spirit Vinegar, Rapeseed Oil, Preservative (Calcium Propionate), Emulsifier (Mono- and Di-Acetyltartaric Esters of Mono- and Di-Glycerides of Fatty Acids), Palm Oil, Flour Treatment Agent (Ascorbic Acid)
	Tesco Finest Super Seeded Bread 800G	Wheat Flour (Wheat Flour, Calcium Carbonate, Iron, Niacin, Thiamin), Water, Mixed Seeds (16%) (Sunflower Seeds, Linseed, Millet, Pumpkin Seed, Poppy Seeds), Mixed Grains (6%) (Malted Wheat Flakes, Oats), Wheat Gluten, Yeast, Salt, Fermented Wheat Flour, Rapeseed Oil, Spirit Vinegar, Emulsifiers (Mono- and Di-Acetyltartaric Esters of Mono- and Di-Glycerides of Fatty Acids, Mono- and Di-Glycerides of Fatty Acids), Palm Oil, Flour Treatment Agent (Ascorbic Acid)
	Tesco Finest Sunflower And Pumpkin Cob Bread 600G	Wheat Flour (Wheat Flour, Calcium Carbonate, Iron, Niacin, Thiamin), Water, Sunflower Seeds (11%), Pumpkin Seed (5%), Wheat Gluten, Yeast, Fermented Wheat Flour, Spirit Vinegar, Salt, Emulsifier (Mono- and Di-Acetyltartaric Esters of Mono- and Di-Glycerides of Fatty Acids), Palm Oil, Rapeseed Oil, Flour Treatment Agent (Ascorbic Acid)
	Morrisons The Best Seeded Loaf 800g	Fortified Wheat Flour (Wheat Flour, Iron, Thiamin, Nicotinic Acid, Calcium Carbonate), Water, Linseed (7%), Sunflower Seeds (3%), Pumpkin Seed (3%), Millet (2%), Poppy Seed (1%), Malted Barley, Salt, Dextrose, Yeast, Emulsifiers (Mono- and Diglycerides of Fatty Acids, Mono- and Diacetyltartaric Acid Esters of Mono- and Diglycerides of Fatty Acids), Caramelised Sugar Powder, Soya Flour, Preservative (Calcium Propionate), Rapeseed Oil, Folic acid
	Burgen Soya & Linseed Loaf 800g	Wheat Flour (with Calcium, Iron, Niacin (B3) and Thiamin (B1)), Water, Linseed, Soya Flour, Cracked Wheat, Kibbled Soya, Wheat Protein, Vegetable Oils (Rapeseed, Sustainable Palm), Yeast, Sugar, Salt, Calcium Carbonate, Vinegar, Emulsifiers: E471, E472e, Flour Treatment Agent: Ascorbic Acid (Vitamin C)

	Warburtons Malted Grain & Seeds 400g	Wheat Flour [with Calcium, Iron, Niacin (B3) and Thiamin (B1)] and Wholemeal Wheat Flour, Water, Malted Wheat Flakes (6%), Wheat Gluten, Yeast, Muscovado Sugar, Wheat Bran, Seed Mix (4%) (Poppy Seed, Millet Seed, Sunflower Seed, Sesame Seed, Brown Linseed), Vegetable Oil (Rapeseed, Sustainable Palm), Salt, Fermented Wheat Starch and Wheat Flour, Soya Flour, Barley Malt Flour, Flour Treatment Agent: Ascorbic Acid (Vitamin C)
	Roberts Bakery Seeded Bloomer 600g	Wheat Flour (with Added Calcium, Iron, Niacin and Thiamine), Water, Seed Blend (15%) (Contains: Sunflower Seeds, Poppy Seeds, Brown Linseeds, Millet and Golden Linseeds), Yeast, Barley Malt Flour, Salt, Emulsifiers: E471, E472E (from Sunflower and Palm), Soya Flour, Preservative: Calcium Propionate (to Inhibit Mould Growth), Rapeseed Oil, Flour Treatment Agent: Ascorbic Acid (Vitamin C)
	Tesco Multiseed Deli Rolls 4 Pack	Wheat Flour (Wheat Flour, Calcium Carbonate, Iron, Niacin, Thiamin), Water, Mixed Seeds (13%) (Brown Linseed, Sunflower Seeds, Millet, Poppy Seeds, Golden Linseed), Palm Oil, Sugar, Yeast, Soya Flour, Salt, Emulsifiers (Mono- and Di-Glycerides of Fatty Acids, Mono- and Di-Acetyltartaric Esters of Mono- and Di-Glycerides of Fatty Acids), Preservative (Calcium Propionate), Flour Treatment Agent (Ascorbic Acid), Wheat Starch
	Morrisons Medium Granary Rolls 4 per pack	Granary Flour (Fortified Wheat Flour (Wheat Flour, Iron, Thiamin, Nicotinic Acid, Calcium Carbonate), Malted Wheat, Wheat Bran, Wheat Gluten), Water, Malted Barley Flour, Fermented Wheat Flour, Rapeseed Oil, Dextrose Monohydrate, Yeast, Emulsifiers (Mono- and Diglycerides of Fatty Acids, Mono- and Diacetyltartaric Acid Esters of Mono and Diglycerides of Fatty Acids, Sodium Stearoyl-2-Lactylate), Salt, Fortified Wheat Flour (Wheat Flour, Iron, Thiamin, Nicotinic Acid, Calcium Carbonate), Soya Flour, Palm Oil, Wheat Flour, Palm Fat, Flour Treatment Agent (Ascorbic Acid), Wheat Starch, Pea Protein, Anti-Caking Agent
	Tesco Seeded Burger Buns	Wheat Flour (Wheat Flour, Calcium Carbonate, Iron, Niacin, Thiamin), Water, Sesame Seeds (7%), Yeast, Emulsifiers (Mono- and Di-Glycerides of Fatty Acids, Sodium Stearoyl-2-Lactylate, Mono- and Di-Acetyltartaric Esters of Mono- and Di-Glycerides of Fatty Acids), Salt, Sugar, Wheat Gluten, Dextrose, Palm Oil, Soya Flour, Thickener (Guar Gum), Rapeseed Oil, Flour Treatment Agents (Ascorbic Acid, L-Cysteine Hydrochloride)
	Sainsbury's Large Multi Seeded Deli Rolls x4	ortified British Wheat Flour (Wheat Flour, Calcium Carbonate, Iron, Niacin, Thiamin), Water, Sunflower Seeds (5%), Brown Linseed (4%), Poppy Seed (3%), Yeast, Rapeseed Oil, Sugar, Whey Powder (Cows' Milk), Salt, Barley Malt Flour, Fermented Wheat Flour, Soya Flour, Palm Oil, Flour Treatment Agent:Ascorbic Acid
Pasta	Tesco Fusilli Pasta Twists 1Kg	Durum Wheat Semolina
	Tesco Everyday Value Penne 500G	Durum Wheat Semolina, Soft Wheat Flour
	Napolina Short Spaghetti 500G	100% Durum Wheat Semolina
	De Cecco Linguine 500G	Durum Wheat Semolina
	Barilla Spaghetti 500G	Durum Wheat Semolina Pasta
	Tesco Pasta Animals 500G	Durum Wheat Semolina, Oat Fibre (8%)

	Tesco Margheritine Soup Pasta 250G	Durum Wheat Semolina
	Tesco Spaghetti 2Kg	Durum Wheat Semolina
	Barilla Fusilli Pasta 500g	Durum Wheat Semolina
	Morrisons Conchiglie 500g	Durum **Wheat** Semolina (100%)
	De Cecco Fusilli 500G	Durum Wheat Semolina
