# Supplementary material for: The Availability and Nutritional Adequacy of Gluten-Free Bread and Pasta
Source: Nutrients. 2018 Sep 25;10(10):1370. doi: 10.3390/nu10101370 (PMC6213709; doi:10.3390/nu10101370)
Supplement: Supplementary file 1 [file nutrients-10-01370-s001.zip › nutrients-354100-supplementary/Supplementary Table 3.pdf]

Supplementary Table 3: Ingredient list for gluten free (GF) bread and pasta products identified in the supermarket survey.

	Product	Ingredients
GF White Products	Tesco Free From White Rolls 4 Pack	Water, Rice Flour, Tapioca Starch, Potato Starch, Bamboo Fibre, Dried Egg White, Psyllium Husk Powder, Rapeseed Oil, Yeast, Sugar, Thickeners (Hydroxypropyl Methyl Cellulose, Xanthan Gum), Maize Starch, Humectant (Glycerol), Salt, Preservative (Calcium Propionate), Maize Flour, Flavouring, Quinoa
	Tesco Free From Sliced White Bread 550G	Water, Rice Flour, Tapioca Starch, Bamboo Fibre, Potato Starch, Yeast, Humectant (Glycerol), Rapeseed Oil, Dried Egg White, Psyllium Husk Powder, Thickeners (Hydroxypropyl Methyl Cellulose, Xanthan Gum), Sugar, Maize Starch, Salt, Preservatives (Calcium Propionate, Sorbic Acid), Maize Flour, Flavouring, Quinoa, Emulsifier (Mono- and Di-Glycerides of Fatty Acids)
	Schar White Ciabatta Rolls 200G	Maize Starch, Water, Maize Flour, Rice Flour, Apple Fibre, Dextrose, Thickener: (Hydroxypropyl Methylcellulose), Sunflower Oil, Soya Protein, Yeast, Salt, Acidifiers: (Tartaric Acid, Citric Acid)
	Ds Wholesome White Sliced Loaf 300G	Maize Starch, Water , Sour Dough (18%): (Rice Flour, Water), Rice Starch, Rice Syrup, Apple Fibre, Sunflower Oil, Millet Flour (2.6%), Soya Protein, Quinoa Flour (1.7%), Thickener: (Hydroxypropyl Methyl Cellulose), Yeast, Salt, Honey (0.5%)
	Genius Gluten Free Toastie 500G	Water, Maize Starch, Potato Starch, Tapioca Starch, Vegetable Oil: Rapeseed Oil, Sunflower Oil, Humectant: Vegetable Glycerol, Psyllium Husk Powder, Yeast, Stabilisers: Hydroxypropyl Methyl Cellulose, Xanthan Gum, Ground Golden Flaxseed, Free Range Dried Egg White, Rice Flour, Dextrose, Iodised Salt: Salt, Potassium Iodate, Fermented Maize Starch, Partially Inverted Sugar Syrup, Calcium Carbonate, Niacin, Iron, Riboflavin, Thiamine, Folic Acid
	Newburn Bakehouse Mini Loaf White 300G	Water, Tapioca Starch, Potato Starch, Maize Starch, Rapeseed Oil, Yeast , Pea Protein, Egg White Powder, Stabiliser: E464, Sugar, Vegetable Fibre (Psyllium), Calcium Carbonate, Fruit Extract (Carob and Apple), Salt, Humectant: Vegetable Glycerine, Preservative: Calcium Propionate (added to inhibit mould growth), Natural Flavouring, Iron
	Warburtons Newburn Bakehouse White Sourdough Artisan	Water , Tapioca Starch , Potato Starch , Maize Starch , Yeast , Rapeseed Oil , Pea Protein , Egg White Powder , Stabiliser: E464 , Gluten Free Sourdough Powder (from Rice) , Sugar , Vegetable Fibre (Psyllium) , Fruit Extract (Carob and Apple) , Salt , Humectant: Vegetable Glycerine , Rice Flour , Preservative: Calcium Propionate (added to inhibit mould growth) , Natural Flavouring
	ASDA Free From White Sliced Loaf	Water , Tapioca Starch , Rice Flour , Rapeseed Oil , Potato Flakes , Psyllium Husk Powder , Humectant (Glycerol) , Sugar , Stabiliser (Hydroxypropyl Methyl Cellulose) , Yeast , Maize Flour , Salt , Fermented Rice Flour .
	Schar Gluten Free Wholesome White Loaf	Maize Starch , Water , Sour Dough (18%): (Rice Flour, Water), Rice Starch, Rice Syrup, Apple Fibre, Sunflower Oil, Millet Flour (2.6%), Soya Protein, Quinoa Flour (1.7%) , Thickener: (Hydroxypropyl Methyl Cellulose) , Yeast , Salt , Honey (0.5%) .

	Genius Gluten Free White Rolls	Water , Maize Starch , Potato Starch , Tapioca Starch , Vegetable Oil: Rapeseed Oil, Sunflower Oil , Humectant: Vegetable Glycerol , Psyllium Husk Powder , Stabilisers: Hydroxypropyl Methyl Cellulose, Xanthan Gum , Yeast , Ground Golden Flaxseed , Free Range Dried Egg White , Concentrated Fruit Juice: Grape , Rice Flour , Dextrose , Rice Starch , Iodised Salt: Salt, Potassium Iodate , Fermented Maize Starch , Partially Inverted Sugar Syrup .
	Genius Gluten Free Soft White Sandwich Loaf	Water , Maize Starch , Potato Starch , Tapioca Starch , Vegetable Oil: Rapeseed Oil, Sunflower Oil , Humectant: Vegetable Glycerol , Psyllium Husk Powder , Yeast , Stabilisers: Hydroxypropyl Methyl Cellulose, Xanthan Gum , Ground Golden Flaxseed , Free Range Dried Egg White , Rice Flour , Dextrose , Iodised Salt: Salt, Potassium Iodate , Fermented Maize Starch , Partially Inverted Sugar Syrup , Calcium Carbonate , Niacin , Iron , Riboflavin , Thiamine , Folic Acid .
	Schar Gluten Free Hamburger Buns	Water , Rice Starch , Rice Flour , Maize Starch , Millet Flour , Sugar , Apple Fibre , Thickener: (Hydroxypropyl Methylcellulose) , Lupine Proteins , Yeast , Sunflower Oil , Salt , Emulsifier: (Mono and Diglycerides of Fatty Acids)
	Schar Gluten Free Panini Rolls	Water , Maize Starch , Maize Flour , Apple Fibre , Rice Flour , Dextrose , Thickener: (Hydroxypropyl Methylcellulose) , Soya Protein , Sunflower Oil , Yeast , Salt , Acids: (Tartaric Acid, Citric Acid)
	Warburtons Gluten Free White Farmhouse Loaf 560g	Water, Tapioca Starch, Potato Starch, Maize Starch, Rapeseed Oil, Yeast, Pea Protein, Egg White Powder, Stabiliser: E464, Sugar, Vegetable Fibre (Psyllium), Calcium Carbonate, Fruit Extract (Carob and Apple), Salt, Humectant: Vegetable Glycerine, Caramelised Sugar, Preservative: Calcium Propionate (added to inhibit Mould growth), Natural Flavouring, Iron
	Warburtons Tiger Artisan Bloomer, Gluten Free 400g	Water, Tapioca Starch, Potato Starch, Maize Starch, Yeast, Rapeseed Oil, Rice Flour Topping (Rice Flour, Sugar, Potato Starch, Flavouring), Pea Protein, Egg White Powder, Stabiliser: E464, Sugar, Vegetable Fibre (Psyllium), Fruit Extract (Carob and Apple), Salt, Humectant: Vegetable Glycerine, Rice Flour, Preservative: Calcium Propionate (added to inhibit mould growth), Natural Flavouring
	Schar Gluten Free White Rolls 348g	Maize Starch, Water, Sour Dough (Rice Flour, Water), Apple Fibre, Millet Flour, Sunflower Oil, Soya Protein, Rice Starch, Yeast, Rice Syrup, Thickener: Hydroxypropyl Methylcellulose, Salt, Sugar
	Morrisons Free From Gluten Free White Bread 350g	Water, Tapioca Starch, Rice Flour, Rapeseed Oil, Potato Flake, Psyllium Husk Powder, Humectant (Glycerol), Stabiliser (Hydroxypropyl Methyl Cellulose), Maize Flour, Sugar, Yeast, Salt, Fermented Rice Flour
	Morrisons Free From Gluten Free 4 White Rolls 4 x 65g	Water, Tapioca Starch, Rice Flour, Rapeseed Oil, Potato Flake, Psyllium Husk Powder, Humectant (Glycerol), Stabiliser (Hydroxypropyl Methyl Cellulose), Maize Flour, Sugar, Yeast, Salt, Fermented Rice Flour
GF Brown Bread	Genius Brown Sliced Bread 535G	Water, Maize Starch, Potato Starch, Tapioca Starch, Vegetable Oil: Rapeseed Oil, Sunflower Oil, Humectant: Vegetable Glycerol, Psyllium Husk Powder, Milled Brown Flaxseed, Stabilisers: Hydroxypropyl Methyl Cellulose, Xanthan Gum, Yeast, Iodised Salt: Salt, Potassium Iodate, Black Treacle, Free Range Dried Egg White, Rice Flour, Dextrose, Fermented Maize Starch, Sugar Beet Fibre, Rice Bran, Calcium Carbonate, Dark Muscovado Sugar, Niacin, Iron, Riboflavin, Thiamine, Folic Acid
	Schar Brown Ciabatta Rolls 200G	Maize Starch, Water, Rice Flour, Sunflower Seeds (7.5%), Buckwheat Flour (7.0%), Linseeds (5.5%), Sugar Beet Syrup, Apple Fibre, Rice Starch, Yeast, Apple Extract, Thickener: (Hydroxypropyl Methyl Cellulose), Soya Protein, Salt, Sunflower Oil, Acidifier: (Tartaric Acid)
	Tesco Free From Brown Bread 550G	Water, Rice Flour, Tapioca Starch, Bamboo Fibre, Potato Starch, Yeast, Rapeseed Oil, Dried Egg White, Psyllium Husk Powder, Buckwheat Flakes (1.5%), Humectant (Glycerol), Thickeners (Hydroxypropyl Methyl Cellulose, Xanthan Gum), Sugar, Concentrated Prune Juice, Rice Bran, Brown Rice

		Flour, Salt, Cocoa Fibre, Preservatives (Calcium Propionate, Sorbic Acid), Flavouring, Emulsifier (Mono- and Di-Glycerides of Fatty Acids)
	Newburn Bakehouse Brown Loaf 560g	Water, Tapioca Starch, Potato Starch, Maize Starch, Rapeseed Oil, Yeast, Pea Protein, Egg White Powder, Stabiliser: E464, Sugar, Vegetable Fibre (Psyllium), Calcium Carbonate, Fruit Extract (Carob and Apple), Salt, Humectant: Vegetable Glycerine, Caramelised Sugar, Preservative: Calcium Propionate (added to inhibit Mould growth), Natural Flavouring, Iron
	ASDA Free From Brown Sliced Loaf	Water , Tapioca Starch , Rice Flour , Rapeseed Oil , Psyllium Husk Powder , Potato Flakes , Treacle , Humectant (Glycerol) , Stabiliser (Hydroxypropyl Methyl Cellulose) , Maize Flour , Yeast , Sugar , Salt , Sugar Beet Fibre , Fermented Rice Flour , Reduced Fat Cocoa Powder
	Schar Gluten Free Brown Ciabatta Rolls	Maize Starch , Water , Rice Flour , Sunflower Seeds (7.5%) , Buckwheat Flour (7.0%) , Linseeds (5.5%) , Sugar Beet Syrup , Apple Fibre , Rice Starch , Yeast , Apple Extract , Thickener: (Hydroxypropyl Methyl Cellulose) , Soya Protein , Salt , Sunflower Oil , Acidifier: (Tartaric Acid) .
GF Seeded	Genius Gluten Free Triple Seeded Farmhouse Bread 535g	Water, Maize Starch, Potato Starch, Mixed Seeds (13%): Sunflower Seeds, Linseed, Millet Seeds, Poppy Seeds, Ground Golden Flaxseed, Milled Brown Flaxseed, Tapioca Starch, Vegetable Oil: Rapeseed Oil, Sunflower Oil, Humectant: Vegetable Glycerol, Black Treacle, Psyllium Husk Powder, Yeast, Stabilisers: Hydroxypropyl Methyl Cellulose, Xanthan Gum, Free Range Dried Egg White, Iodised Salt: Salt, Potassium Iodate, Rice Flour, Dextrose, Fermented Maize Starch, Sugar Beet Fibre, Rice Bran, Calcium Carbonate, Dark Muscovado Sugar, Niacin, Iron, Riboflavin, Thiamine, Folic Acid
	Schar Wholesome Seeded Sliced Bread 300G	Water, Maize Starch, Sour Dough (16%): (Rice Flour, Water), Rice Starch, Cereals (4, 3%): [Millet Flour (2.6%), Quinoa Flour (1.7%)], Apple Fibre, Sugar Beet Syrup, Sunflower Oil, Rice Syrup, Soya Flakes (2.1%), Sunflower Seeds (2.1%), Soya Bran (1.9%), Linseeds (1.9%), Thickener: (Hydroxypropyl Methyl Cellulose), Millet Flakes (1.4%), Soya Protein, Yeast, Sea Salt, Honey (0.5%)
	Tesco Free From Sliced Seeded Bread 550G	Water, Rice Flour, Mixed Seeds (11%), Tapioca Starch, Bamboo Fibre, Potato Starch, Yeast, Rapeseed Oil, Dried Egg White, Psyllium Husk Powder, Humectant (Glycerol), Thickeners (Hydroxypropyl Methyl Cellulose, Xanthan Gum), Sugar, Salt, Concentrated Prune Juice, Preservatives (Calcium Propionate, Sorbic Acid), Emulsifier (Mono- and Di-Glycerides of Fatty Acids). Mixed Seeds contains: Sunflower Seeds, Brown Linseed, Golden Linseed, Poppy Seeds, Millet Seeds
	Genius Gluten Free Wholesome Five Seeded 535G	Water, Maize Starch, Potato Starch, Mixed Seeds (13%): Sunflower Seeds, Linseed, Millet Seeds, Poppy Seeds, Ground Golden Flaxseed, Milled Brown Flaxseed, Tapioca Starch, Vegetable Oil: Rapeseed Oil, Sunflower Oil, Humectant: Vegetable Glycerol, Black Treacle, Psyllium Husk Powder, Yeast, Stabilisers: Hydroxypropyl Methyl Cellulose, Xanthan Gum, Free Range Dried Egg White, Iodised Salt: Salt, Potassium Iodate, Rice Flour, Dextrose, Fermented Maize Starch, Sugar Beet Fibre, Rice Bran, Calcium Carbonate, Dark Muscovado Sugar, Niacin, Iron, Riboflavin, Thiamine, Folic Acid
	Tesco Free From Multiseed Slice Bread 400G	Water, Mixed Seeds (11%), Modified Maize Starch, Modified Tapioca Starch, Rice Flour, Modified Potato Starch, Rapeseed Oil, Psyllium Husk Powder, Prunes, Maize Starch, Yeast, Maize Flour, Honey, Bamboo Fibre, Thickener (Hydroxypropyl Methyl Cellulose), Salt, Rice Starch, Glucose Syrup, Buckwheat Flour, Preservative (Calcium Propionate), Flax Seeds, Emulsifier (Xanthan Gum), Corn Syrup, Lactobacillus Culture, Yeast Powder, Enzymes, Mixed Seeds contain: Sunflower Seeds, Brown Linseed, Golden Linseed, Millet, Poppy Seeds
	Schar Wholesome Vitality Loaf Gluten Free 350G	Sour Dough 25% (Rice Flour, Water), Maize Starch, Water, Rice Flour, Sunflower Seeds 6, 5%, Linseeds 6, 5%, Apple Fibre, Quinoa Flour 2, 6%, Apple Extract, Yeast, Sorghum Flour 2, 4%, Sunflower Oil, Thickeners: Hydroxypropyl Methyl Cellulose, Chestnut Flour 1, 0%, Salt, Caramelised Sugar
	Tesco Free From Brown Seeded Rolls 4 Pack	Water, Rice Flour, Mixed Seeds (10%), Tapioca Starch, Potato Starch, Bamboo Fibre, Psyllium Husk Powder, Dried Egg White, Rapeseed Oil, Yeast, Thickeners (Hydroxypropyl Methyl Cellulose, Xanthan Gum), Sugar, Concentrated Prune Juice, Salt, Cocoa Fibre, Preservative (Calcium

		Propionate). Mixed Seeds contain: Brown Linseed, Sunflower Seeds, Golden Linseed, Millet Seeds, Poppy Seeds
	Genius Multiseed Rolls 4 Pack	Water, Maize Starch, Potato Starch, Mixed Seeds (11%): Sunflower Seeds, Linseeds, Millet Seeds, Poppy Seeds, Tapioca Starch, Vegetable Oil: Rapeseed Oil, Sunflower Oil, Black Treacle, Humectant: Vegetable Glycerol, Psyllium Husk Powder, Yeast, Stabilisers: Hydroxypropyl Methyl Cellulose, Xanthan Gum, Sugar Beet Fibre, Rice Bran, Milled Brown Flaxseed, Free Range Dried Egg White, Concentrated Fruit Juice: Grape, Rice Flour, Iodised Salt: Salt, Potassium Iodate, Rice Starch, Fermented Maize Starch
	Newburn Bakehouse Mini Loaf Seeded 300G	Water, Tapioca Starch, Seed Mix (8%) (Sunflower Seed, Linseed, Millet Speed, Poppy Seed), Potato Starch, Maize Starch, Rapeseed Oil, Yeast, Pea Protein, Egg White Powder, Stabiliser: E464, Sugar, Vegetable Fibre (Psyllium), Calcium Carbonate, Fruit Extract (Carob and Apple), Salt, Humectant: Vegetable Glycerine, Preservative: Calcium Propionate (added to inhibit mould growth), Natural Flavouring, Caramelised Sugar, Iron
	Burgen Free From Sunflower And Chia Seed Bread 500G	Water, Rice Flour, Tapioca Starch, Mixed Seeds (11%) (Sunflower Seeds, Chia Seeds, Brown Linseed), Potato Starch, Rapeseed Oil, Yeast, Maize Flour, Rice Starch, Psyllium Fibre, Humectant: Glycerol, Free Range Egg White Powder, Sugar, Stabilisers: E464, Xanthan Gum, Salt, Cornflour, Sugar Beet Fibre, Fermented Rice Flour
	Bfree Brown Seeded Bloomer High Fibre 400G	Water, Potato Flour, Corn Starch, Tapioca Starch, Brown Rice Flour, Buckwheat Flour, Sunflower Seeds 3.5%, Thickening Agent (Xanthan Gum, Cellulose, Agar Agar), Treacle, Linseeds 1.5%, Glycerol, Yeast, Rice Bran, Pea Protein, Rapeseed Oil, Salt, Apple Fibre, Sourdough (Fermented Quinoa, Rice and Maize Flour), Psyllium Husk, Acids (Citric Acid, Malic Acid, Tartaric Acid), Acidifier (Glucono-Delta-Lactone), Flour Treatment Agent (Ascorbic Acid)
	Schar Seeded Ciabatta Gluten Free 210G	Sourdough (Rice Flour, Water), Maize Starch, Water, Sunflower Seeds (12%), Linseed (10%), Rice Flour, Buckwheat Flour (6%), Apple Fibre, Rice Starch, Yeast, Apple Extract, Sugar Beet Syrup, Thickener: Hydroxypropyl Methylcellulose, Soya Protein, Sunflower Oil, Salt, Caramelised Sugar, Acid: Tartaric Acid
	Tesco Free From Ancient Grain Cob 400G	Water, Rice Flour, Tapioca Starch, Hulled Buckwheat Grains (5%), Potato Starch, Dried Egg White, Bamboo Fibre, Rapeseed Oil, Yeast, Psyllium Husk Powder, Sugar, Maize Starch, Thickeners (Hydroxypropyl Methyl Cellulose, Xanthan Gum), Amaranth Seeds (1%), Rice Bran, Red Quinoa (1%), Salt, Concentrated Prune Juice, Teff Grains (0.5%), Preservatives (Calcium Propionate, Potassium Sorbate), Pumpkin Seeds, Golden Linseed, Sunflower Seeds, Flavouring
	Kelkin Sourdough Multiseed 200G	Sourdough (41%) [Water, Rice Flour, Maize Starch, Buckwheat Flour, Salt, Culture Starter], Water, Potato Starch, Whey (Milk) Powder, Linseeds (5%), Sunflower Seeds (3%), Maize Starch, Sunflower Oil, Inulin, Yeast, Soya Protein Isolate, Sugar, Thickeners: Xanthan Gum & Guar Gum, Poppy Seeds (2%), Olive Oil (2%), Glucose-Fructose Syrup, Emulsifiers: Soya Lecithin & Diacetyltartaric Acid Esters of Mono- and Diglycerides, Humectant: Glycerine, Salt
	Morrisons Free From Gluten Free Seeded Bread 350g	Water, Tapioca Starch, Rice Flour, Seeds (6%) (Pumpkin, Linseed, Quinoa, Poppy), Rapeseed Oil, Potato Flake, Psyllium Husk Powder, Humectant (Glycerol), Stabiliser (Hydroxypropyl Methyl Cellulose), Maize Flour, Sugar, Yeast, Salt, Fermented Rice Flour
	Warburtons Seeded Farmhouse Loaf Gluten Free 560g	Water, Tapioca Starch, Seed Mix (8%) (Sunflower Seed, Linseed, Millet Seed, Poppy Seed), Potato Starch, Maize Starch, Rapeseed Oil, Yeast, Pea Protein, Egg White Powder, Stabiliser: E464, Sugar, Vegetable Fibre (Psyllium), Calcium Carbonate, Fruit Extract (Carob and Apple), Salt, Humectant: Vegetable Glycerine, Preservative: Calcium Propionate (added to inhibit mould growth), Caramelised Sugar, Natural Flavouring, Iron
GF Pasta	Morrisons Free From Fusilli 500g	Cornflour (69%), Rice Flour (17%), Water, Emulsifier (Mono- and Diglycerides of Fatty Acids)

	Morrisons Free From Green Pea Pasta 250g	Pea flour (87%), Water
	Morrisons Free From Dinosaurs Pasta 250g	Cornflour (80%), Rice Flour, Emulsifier (Mono- and Diglycerides of Fatty Acids)
	Morrisons Free From Red Lentil Pasta 250g	Red Lentils (87%), Water
	Explore Organic Edamame Spaghetti 200g	Organic Edamame Beans (Green Soybeans)
	Explore Cusine Organic Edamame & Mung Bean Fettuccine 200g	Organic Edamame Beans (Green Soybeans) (80%), Organic Mung Beans (20%)
	Sainsbury's Deliciously Freefrom Fusilli 500g	Rice Flour (40%), Wholegrain Rice Flour (31%), Maize Flour (26%), Quinoa Flour (3%), Emulsifier: Mono- and Diglycerides of Fatty Acids.
	Doves Farm Gluten Free Organic Penne Pasta 500g	Maize Flour* 70%, Rice Flour 30%
	Sainsbury's Buckwheat Penne 500g	100% Buckwheat Flour
	Napolina Gluten Free Green Pea Fusilli 250g	Green Pea Flour
	Sainsbury's Deliciously Freefrom Spaghetti 500g	Rice Flour (40%), Wholegrain Rice Flour (31%), Maize Flour (26%), Quinoa Flour (3%), Emulsifier: Mono- and Diglycerides of Fatty Acids.
	Barilla Gluten Free Spaghetti 400G	White Corn Flour (65%), Yellow Corn Flour (29.5%), Rice Flour (5%), Water, Emulsifier: Mono- and Diglycerides of Fatty Acids
	Tesco Free From Penne Pasta 500G	Rice Flour, White Maize Flour, Yellow Maize Flour
	Tesco Free From Lasagne Sheets 250G	Rice Flour, White Maize Flour, Yellow Maize Flour
	Sainsbury's Deliciously Freefrom Penne 500g	Rice Flour (40%), Wholegrain Rice Flour (31%), Maize flour (26%), Quinoa Flour (3%), Emulsifier: Mono- and Diglycerides of Fatty Acids.
	Morrisons Free From Fusilli 500g	Cornflour (69%), Rice Flour (17%), Water, Emulsifier (Mono- and Diglycerides of Fatty Acids)
